# CRISPR/Cas12a-Chemiluminescence Cascaded Bioassay for Amplification-Free and Sensitive Detection of Nucleic Acids

**DOI:** 10.3390/bios15080479

**Published:** 2025-07-24

**Authors:** Xiaotian Guan, Peizheng Wang, Yi Wang, Shuqing Sun

**Affiliations:** 1 Institute of Biopharmaceutical and Healthcare Engineering, Shenzhen International Graduate School, Tsinghua University, Shenzhen 518055, China; 2 Experimental Research Center, China Academy of Chinese Medical Sciences, Beijing 100010, China

**Keywords:** CRISPR/Cas12a, chemiluminescence, amplification-free, nucleic acid detection, human papillomavirus type 16 (HPV-16), parvovirus B19 (PB-19)

## Abstract

The CRISPR/Cas system has attracted increasing attention in accurate nucleic acid detection. Herein, we reported a CRISPR/Cas12a-chemiluminescence cascaded bioassay (CCCB) for the amplification-free and sensitive detection of human papillomavirus type 16 (HPV-16) and parvovirus B19 (PB-19). A magnetic bead (MB)-linking single-stranded DNA (LssDNA)-alkaline phosphatase (ALP) complex was constructed as the core component of the bioassay. During the detection process, the single-stranded target DNA was captured and enriched by LssDNA and then activated the trans-cleavage activity of Cas12a. Due to the Cas12a-mediated cleavage of LssDNA, ALP was released from the MB, subsequently catalyzing the substrate to generate a chemiluminescence (CL) signal. Given the cascade combination of CRISPR/Cas12a with the CL technique, the limits of detection for HPV-16 and PB-19 DNA were determined as 0.14 pM and 0.37 pM, respectively, and the whole detection could be completed within 60 min. The practicality and reliability of the platform were validated through target-spiked clinical specimens, and the recovery rate was 93.4–103.5%. This dual-amplification strategy—operating without target pre-amplification—featured high specificity, low contamination risk, facile preparation, and robust stability. It provides a novel approach for sensitive nucleic acid detection, with the potential for rapid extension to the diagnosis of various infectious diseases.

## 1. Introduction

Nucleic acid detection plays a crucial role in the prevention and control of infectious diseases, which can quickly identify infected individuals for accurate isolation and etiological treatment [1,2]. Among these sources of infection, human papillomavirus type 16 (HPV-16) is a high-risk DNA virus, and most cervical cancer and its precursors are caused by persistent high-risk human papillomavirus infection [3,4]. Human parvovirus B19 (PB-19) is another DNA virus. It commonly infects children and pregnant women. Transmission occurs via respiratory secretions, hand-to-mouth contact, blood transfusion, or transplacental transmission. This may cause severe outcomes including infectious erythema, joint pain, fetal death, or miscarriage [5,6]. Nucleic acid, as the genetic material of viruses, has the advantages of high specificity, easy accessibility, and wide applicability in pathogen identification. Therefore, it is urgent and necessary to develop sensitive and reliable nucleic acid detection methods for the accurate diagnosis of these infectious viruses.

Currently, the most widely used nucleic acid detection technology is based on the target amplification strategy, among which the polymerase chain reaction (PCR) is regarded as the gold standard [7]. The PCR method achieves cycles of DNA denaturation, primer annealing, and extension through temperature control, so as to amplify specific DNA fragments, which offers high sensitivity and specificity [8,9,10,11]. For example, Taherkhani et al. utilized PCR to conduct a statistical analysis of the prevalence, genotype pattern, and risk factors of PB-19 infection among a population-based sample of pregnant women [12]. Although it provides an effective tool for nucleic acid analysis, most of these amplification-based strategies not only increase the risk of contamination, but also involve well-trained personnel and complex instruments, and may lead to false-positive results during the amplification process [13,14]. In a previous study, Wang et al. compared the clinical application of amplification-based and amplification-free metagenomic next-generation sequencing (mNGS) in infectious diseases, and demonstrated that the amplification-free approach reduced false-positive results [15]. Accordingly, recent studies have developed advanced nucleic acid analysis platforms without target amplification to break the limitations of traditional methods [16,17,18]. For instance, Xi et al. reported a gel electrophoresis platform with a catalytic hairpin assembly (CHA) as a signal amplification module for the reliable detection of DNA and miRNA [16]. Nevertheless, these emerging methods still face challenges such as insufficient sensitivity, prolonged detection time, and laborious procedures, making them unsuitable for real clinical application.

Clustered regularly interspaced short palindromic repeats (CRISPR), originally a commonly used gene-editing technology, has recently been developed as a powerful tool for biomarker assays due to its signal-amplification effect [19,20,21]. Among them, the CRISPR/Cas12a system has been applied to nucleic acid detection because of its trans-cleavage activity toward the non-target single-stranded nucleic acids nearby via the recognition of trigger DNA [22,23]. Cas12a can be activated by both single-stranded DNA (ssDNA) and double-stranded DNA (dsDNA), though activation by dsDNA requires the protospacer adjacent motif (PAM) of the “TTTN” sequence [24]. In addition, owing to the programmability of CRISPR-guided RNA (crRNA), this system is easily tailored to analyze specific target sequences [25,26]. A typical example is that Chen et al. created a method termed DNA endonuclease-targeted CRISPR trans reporter (DETECTR) by combining Cas12a with isothermal amplification, which achieved high sensitivity for DNA detection [27]. A chemiluminescence (CL) assay is another well-established and sensitive bioanalytical methodology that employs enzymes to catalyze substrates, thereby emitting photons through the chemical reaction and converting the target analyte concentration into a measurable optical signal [28,29,30]. For example, Liu et al. constructed an alkaline phosphatase (ALP)-based CL biosensor for poly (ADP-ribose) polymerase-1 (PARP-1) detection with a limit of detection (LOD) of 2.94 × 10^−7^ U/μL [31]. Inspired by ALP as a catalytic amplifier, Chen et al. recently developed a split-type ratiometric fluorescent assay for H1N1 DNA detection, achieving a LOD of 10 pM [32]. However, conventional single-stage amplification approaches often suffer from limited sensitivity compared to cascade systems [32,33]. Consequently, integrating CRISPR/Cas12a with CL to achieve cascaded signal amplification would represent an attractive non-amplification strategy for nucleic acid analysis.

In this work, we designed a CRISPR/Cas12a-chemiluminescence cascaded bioassay (CCCB) for the amplification-free and sensitive detection of HPV-16 and PB-19. A magnetic bead (MB)-linking ssDNA (LssDNA)-ALP complex (MB-LssDNA-ALP) was constructed as the core component of this biosensing platform. Within the detection workflow, the single-stranded target DNA was first captured and enriched by LssDNA through base hybridization. Cas12a’s trans-cleavage activity was then activated via sequence recognition between crRNA and target DNA. Subsequently, efficient Cas12a-mediated cleavage of LssDNA released ALP from MB into solution. Finally, ALP catalyzed the CL substrate to generate signals for the quantitative determination of target DNA. This CCCB platform avoids the pre-amplification process in traditional nucleic acid assays through the cascade of CRISPR/Cas12a and CL, achieving high sensitivity while effectively reducing the risk of contamination, and has been proven to be reliable for clinical specimen measurement. Furthermore, characterized by high specificity, rapid testing, robust stability, and minimal instrumentation requirements, it holds significant promise for the accurate diagnosis of various infectious diseases.

## 2. Materials and Methods

### 2.1. Materials and Reagents

Carboxylated magnetic beads (MyOne^TM^) were obtained from Thermo Fisher Scientific (Waltham, MA, USA). 2-Morpholinoethanesulphonic acid (MES) and 1-(3-Dimethylaminopropyl)-3-ethylcarbodiimide hydrochloride (EDC) were bought from Aladdin Chemistry Co., Ltd. (Shanghai, China). Commercialized 3-[2-spiroadamatane]- 4-methoxy-4-[3-phosphoryloxy]-phenyl-1,2-dioxetane) (AMPPD) substrate solution was obtained from Mindray Co., Ltd. (Shenzhen, China). Bovine serum albumin (BSA) and Tris-HCl were purchased from Beyotime Co., Ltd. (Shanghai, China). Cas12a enzymes, streptavidin (SA)-alkaline phosphatase, and all oligonucleotides were purchased from Sangon Biotech Co., Ltd. (Shanghai, China). The oligonucleotide sequences used in this work were designed according to the previous study and were listed in Table S1 [34].

### 2.2. Preparation and Characterization of MB-Linking ssDNA-ALP

Firstly, magnetic bead-linking single-stranded DNA complexes (MB-LssDNA) were prepared by the reaction of amino and carboxyl groups to form amide bonds. Specifically, 1 mg MB was suspended in 300 μL MEST buffer (100 mM MES, 0.5 mg/mL Tween 20, pH = 4.8), followed by adding 100 μL EDC solution (10 mg/mL, ultra-pure water (UP)) and 20 μL NH_2_-modified LssDNA solution (UP). After mixing, the reaction was carried out overnight at 30 r/min on a rotating mixer (JOANLAB, RMO-80PRO, Huzhou, China) at room temperature. Subsequently, the MB component was collected and then incubated with 500 μL TT buffer (250 mM Tris-HCl, 10 mg/mL BSA, 0.1 mg/mL Tween20, pH = 8.0) for 30 min followed by magnetic separation, repeating this process three times to obtain MB-LssDNA. Next, to prepare MB-LssDNA-ALP, the MB-LssDNA was then transferred to 400 μL Tris-HCl buffer (10 mM Tris-HCl, 50 mM NaCl, 15 mM MgCl_2_, 0.1 mg/mL BSA, 0.5 mg/mL Tween20, pH = 7.8), and 40 μL SA-labeled ALP (0.01 mg/mL, Tris-HCl buffer) was added. The mixture was incubated at 37 °C for 30 min, during which ALP was linked to LssDNA through the binding of streptavidin and biotin to obtain MB-LssDNA-ALP. The MB-LssDNA-ALP was washed three times via magnetic separation with 1 mL Tris-HCl buffer to remove unreacted substances, and diluted to 3 mL with Tris-HCl buffer for future use.

The components for the bioassay were characterized by zeta potential and polydispersity index (PDI) using a Zetasizer Nano ZS (Malvern, Malvern, UK). The conjugation efficiency of LssDNA was quantitatively analyzed by measuring the fluorescence (FL) signal of Cy3-LssDNA in the supernatant after synthesizing the MB-LssDNA using Cy3-labeled sequences.

### 2.3. Procedure of Nucleic Acid Detection

MB-LssDNA-ALP (150 μL) was mixed with the sample (50 μL) and reacted at 37 °C for 15 min followed by magnetic separation to capture the target nucleic acids. Subsequently, 106 μL crRNA/Cas12a (crRNA and Cas12a were pre-mixed using Tris-HCl at a 1:1 molar ratio for 5 min) were added to form activated crRNA/Cas12a-target nucleic acid complexes and arbitrarily cut LssDNA at 37 °C for 30 min. Next, 30 μL of supernatant containing released ALP was mixed with 170 μL AMPPD and then transferred into a 96-well plate. After incubation for 15 min, the CL intensity (λ_em_ = 548 nm) was measured with a microplate reader (Infinite M1000 PRO, Tecan, Männedorf, Switzerland).

### 2.4. Clinical Sample Collection and Processing

All plasma specimens from healthy donors were collected from the Department of Laboratory Medicine, Shenzhen Second People’s Hospital, Shenzhen, China. The plasma specimens were centrifuged at 3000× *g* for 15 min to remove platelets. The supernatants were carefully collected and diluted 10 times with Tris-HCl buffer for testing.

### 2.5. Data Analysis and Statistical Testing

The results are reported as the mean ± standard error of the mean. Significant differences were analyzed using Student’s *t*-test: * *p* < 0.05, ** *p* < 0.01, *** *p* < 0.001, **** *p* < 0.0001, and ns: no significant difference. All data were obtained from three independent experiments with similar results unless noted otherwise.

## 3. Results and Discussion

### 3.1. Principle of CCCB for Nucleic Acid Detection

**Figure 1:**
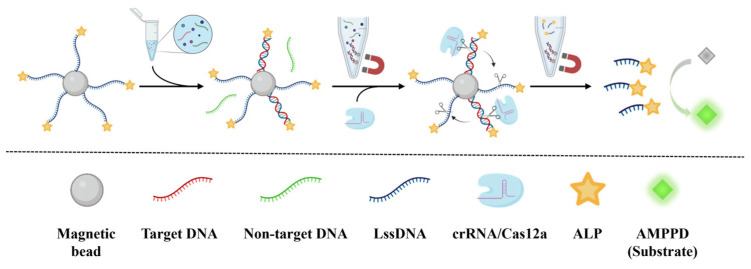
Schematic illustration of the CCCB strategy for nucleic acid detection.

The principle of CCCB for the amplification-free and sensitive detection of nucleic acids is shown in Figure 1. The MB-LssDNA-ALP complex was constructed as the core component of this biosensing strategy. During the detection process, the single-stranded target DNA is initially captured and enriched by LssDNA via base hybridization to form a double-stranded structure. Unreacted substances in the sample are washed away by magnetic separation. Subsequently, crRNA/Cas12a is added, and the trans-cleavage activity of Cas12a is then activated through sequence recognition between crRNA and double-stranded target DNA containing a PAM sequence. Following the efficient Cas12a-mediated cleavage of LssDNA, ALP is released from MB into solution. After magnetic separation and collecting the supernatant containing free ALP, the ALP catalyzes AMPPD to generate a CL signal, which can be used for the quantitative measurement of the target DNA in the sample. Therefore, enhanced signal amplification of the target nucleic acids is achieved through a cascaded enzymatic reaction that integrates the trans-cleavage activity of Cas12a with the catalytic function of ALP. Based on the principle, two LssDNA sequences were designed for the detection of HPV-16 and PB-19 to demonstrate the generality of this CCCB strategy.

### 3.2. Characterization of MB-LssDNA

**Figure 2:**
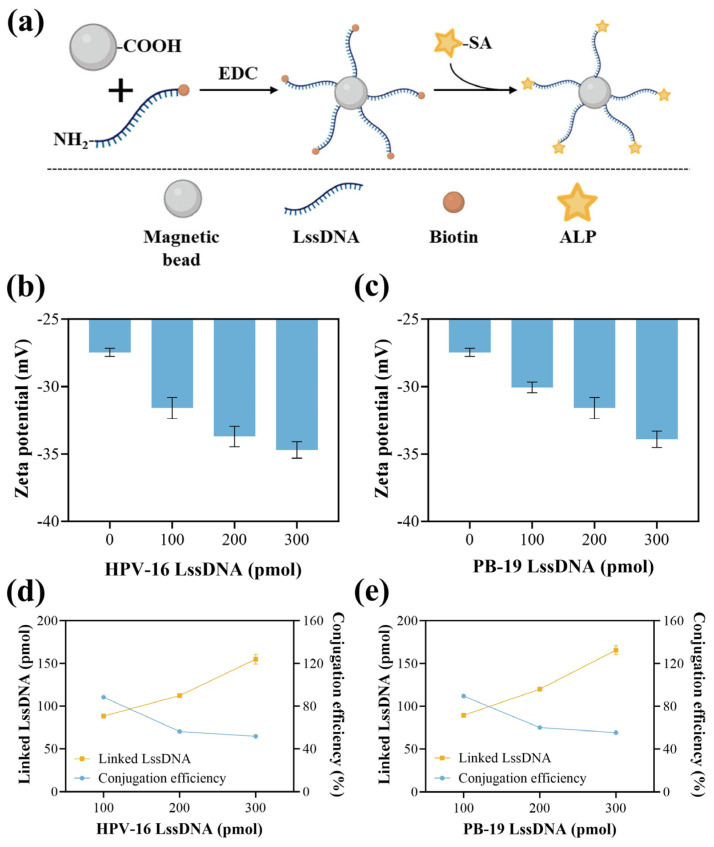
Preparation principle and characterization of the bioassay components used in this work. (**a**) Preparation principle of MB-LssDNA-ALP; zeta potential results of (**b**) MB-LssDNA(HPV-16) and (**c**) MB-LssDNA(PB-19). The quantities and conjugation efficiencies of (**d**) HPV-16 and (**e**) PB-19 LssDNA conjugated to MB. The linked LssDNA = total LssDNA input − unlinked LssDNA in the supernatant; the conjugation efficiency = (linked LssDNA/total LssDNA input) × 100%.

The preparation principle of the MB-LssDNA-ALP is illustrated in Figure 2a, which involves the prior synthesis of MB-LssDNA. Therefore, the synthesis of MB-LssDNA was first demonstrated. With the increasing proportion of LssDNA, the zeta potential of MB-LssDNA decreased from approximately −27 to −35 mV, indicating that more LssDNA combined with the magnetic beads (Figure 2b,c). The particle size of the complexes was measured to be 1221 ± 123 nm, and the PDI was below 0.2, proving the monodispersity of MB-LssDNA (Figure S1). The number of LssDNA linked to MB is crucial for the capture of target DNA in the sample. Hence, quantitative analysis was conducted on the number of combined LssDNA by detecting the Cy3-labeled LssDNA in the supernatant after the linking process. The concentration standard curve of Cy3-labeled LssDNA is shown in Figure S2. As shown in Figure 2d,e, for both HPV-16 and PB-19 detection systems, the optimal condition was selected as 200 pmol LssDNA, due to the acceptable linked LssDNA number (≈116 pmol) and appropriate conjugation efficiency (≈58%) under this condition, as well as the lower material cost compared to 300 pmol. Thus, the optimized MB-LssDNA complex was obtained.

### 3.3. Feasibility Verification

**Figure 3:**
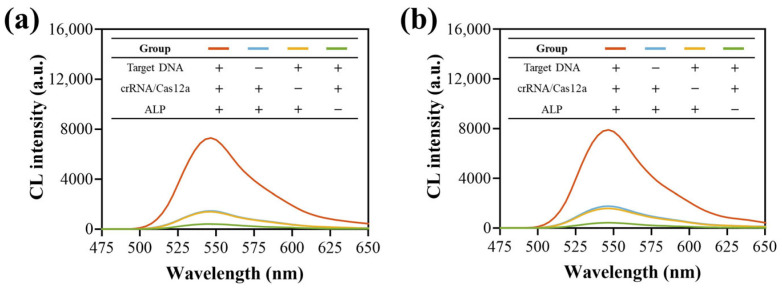
Feasibility verification of the CCCB strategy. The influence of different components on (**a**) HPV-16 and (**b**) PB-19 detection. The concentration of target DNA was set to 1 nM.

To verify the feasibility of the CCCB method, the influence of different components on HPV-16 (Figure 3a) and PB-19 (Figure 3b) detection was studied using the developed MB-LssDNA-ALP and Tris-HCl buffer sample. The CL intensity increased upon target DNA introduction, while the absence of either target DNA or crRNA/Cas12a yielded comparably weak CL signals, which may be due to the presence of trace amounts of MB-LssDNA-ALP in the supernatant after the final magnetic separation, catalyzing the substrate and producing this background signal. Notably, ALP absence significantly decreased the background noise, supporting this hypothesis and also proving that ALP was linked to MB-LssDNA. These results demonstrated the platform’s feasibility for both HPV-16 and PB-19 detection.

### 3.4. Optimization of Experimental Conditions

To achieve high sensitivity with the CCCB strategy, the experimental conditions were optimized. The optimization of analysis conditions for HPV-16 was shown in Figure S3. In order to maximize the signal-to-noise (S/N) ratio, the concentrations of crRNA/Cas12a and Mg^2+^ in the detection procedure were chosen to be 30 nM and 15 nM, respectively. The pH of the Tris-HCl buffer was set to 7.8. In this method, LssDNA served three functions: linking ALP to MB, capturing target DNA, and being cleaved by Cas12a, all critical for detection performance, and thus the sequence length of LssDNA was investigated. Figure S3d demonstrates a higher S/N ratio with a shorter LssDNA sequence; therefore, a length of 39 nt was considered the optimum. This may be due to the Cas12a repeatedly cutting the same long sequence of LssDNA multiple times, releasing less ALP than short LssDNA, and resulting in a lower S/N ratio. As shown in Figure S4, the experimental conditions for PB-19 detection followed a similar rule as for HPV-16 testing, so the same parameters as HPV-16 testing were selected for PB-19 detection to achieve ideal analysis performance.

### 3.5. Key Performance Verification

**Figure 4:**
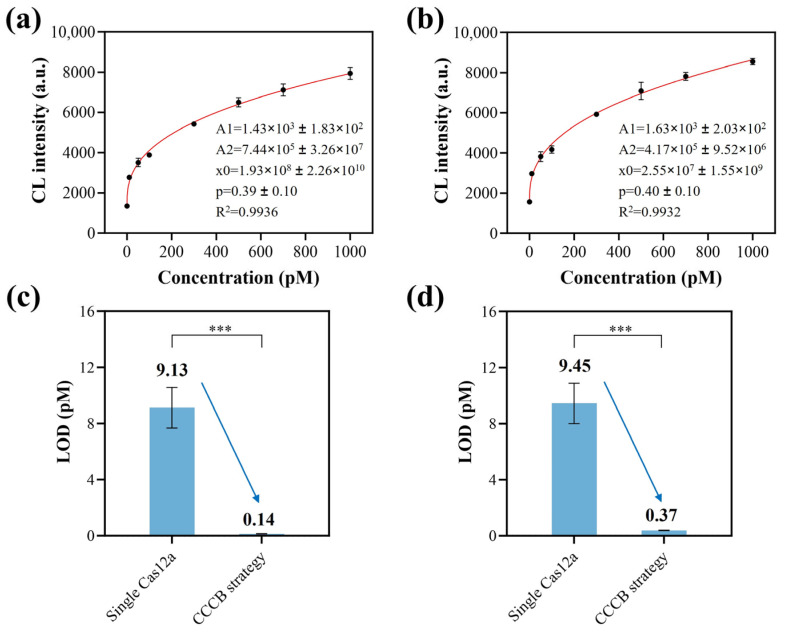
Sensitivity evaluation of the CCCB strategy. The concentration standard curve for (**a**) HPV-16 and (**b**) PB-16 detection. The target DNAs were 0, 10, 50, 100, 300, 500, 700, and 1000 pM. The four-parameter logistic fit equation was *y* = A2 + (A1 − A2)/(1 + (*x*/x0)^p). Comparison of LODs between the CCCB strategy and the single Cas12a-based detection system for (**c**) HPV-16 and (**d**) PB-19 analysis.

With the aim of evaluating the analytical performance of the proposed CCCB platform, the sensitivity was verified first. Under the above optimized experimental conditions, as shown in Figure 4a,b, the relationship between the CL intensity and the concentration of target DNA fitted to the four-parameter logistic fit within the range from 10 to 1000 pM for both HPV-16 (R^2^ = 0.9936) and PB-19 (R^2^ = 0.9932) analysis. The LODs of HPV-16 and PB-19 were calculated to be 0.14 and 0.37 pM according to the 3σ criterion, respectively. It is worth noting the high uncertainties of the parameters A2 and x0. This may be because the fitting points used in this work were all distributed in the low value range of the whole fitting curve, thus leading to a high uncertainty for fitting the high value range of the curve. It is still reliable to calculate values within the fitting range by using the established calibration curve. To demonstrate the superiority of the cascaded enzymatic strategy constructed by this work, we compared the LOD of the CCCB platform with that of the single enzymatic detection system. The principle of the single Cas12a-based detection system was illustrated in Figure S5, in which Cy3 was used instead of ALP. The concentration standard curves of the single Cas12a-based method were shown in Figure S6. Figure 4c,d showed that the LODs based on single Cas12a were 9.13 pM (HPV-16) and 9.45 pM (PB-19). Thus, the sensitivity of the cascaded enzymatic strategy was increased by 65 times and 25 times in the detection of HPV-16 and PB-19, respectively, compared to the single enzymatic method. In addition, compared with previous studies (Table S2), the CCCB method achieved high sensitivity within a short testing time, making it suitable for practical application.

**Figure 5:**
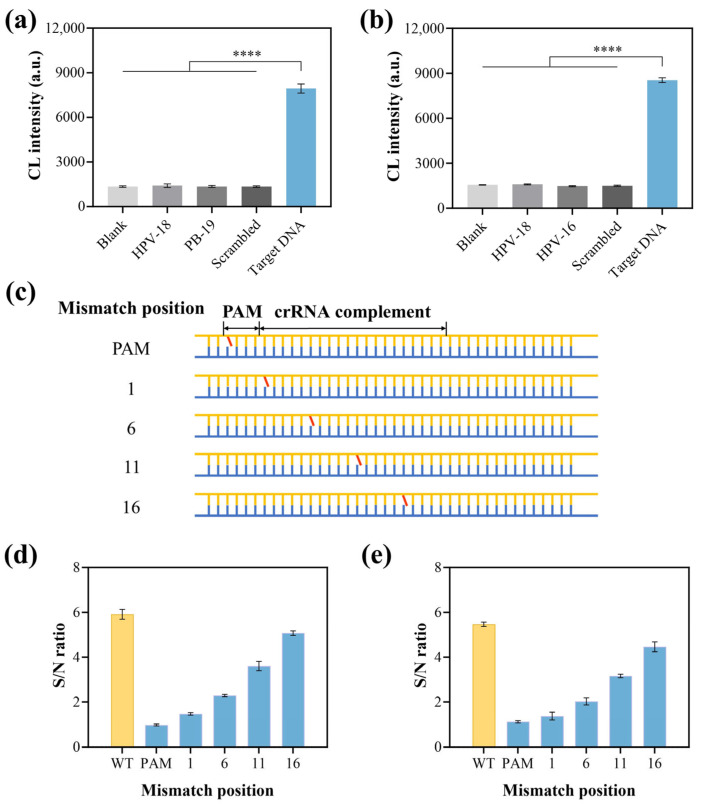
Specificity study of the CCCB strategy. Specificity verification of (**a**) HPV-16 and (**b**) PB-19 detection. (**c**) Target DNA with mismatches at different positions, including PAM regions and crRNA complements at different positions: 1, 6, 11, and 16. Evaluation of the influence of mismatches at different positions on the CCCB signal for (**d**) HPV-16 and (**e**) PB-19 analysis. A target concentration of 1 nM was applied for all the groups (non-target, WT, and mismatched DNA).

The specificity of the CCCB platform was then verified. HPV-18, PB-19, and a scrambled sequence were applied to evaluate the specificity for HPV-16 detection. As shown in Figure 5a, the non-target sequences demonstrated signal changes that were comparable to that of the blank group, proving a good specificity of the LssDNA and crRNA/Cas12a in differentiating HPV-16 from non-targets. A similar result was obtained for PB-19 testing (Figure 5b). Furthermore, as a biosensing platform, distinguishing mismatches in nucleic acid–base pairs is important for the potential application of identifying disease-related point mutations [34,35]. Therefore, the CCCB was challenged by mismatched nucleic acid targets with artificial mutations at different positions (Figure 5c). Figure 5d,e showed that mutations in the PAM region led to a complete diminishment of the CL signal compared with the wild-type (WT) target sequence. As the mutation site moved away from the PAM region, the CL signal gradually recovered. This phenomenon indicated that the PAM sequence was mandatory for crRNA/Cas12a to recognize the double-stranded target and then be activated for trans-cleavage activity, which was consistent with previous studies [27,36]. Due to the significant differentiation of signals from different mismatched positions, the CCCB platform has the potential to identify base mutations in target sequences. Overall, this method showed good specificity in nucleic acid detection.

Excellent repeatability is the prerequisite for a high-accuracy analysis platform. As shown in Figure S7, the repeatability of this method was investigated through repeated testing of identical samples at different concentrations. The CL intensity exhibited no statistically meaningful variation across five replicates at identical concentrations for the analysis of both HPV-16 and PB-19. The relative standard deviation (RSD) of all test results for the same concentration sample was less than 4.1%. This good repeatability may be attributed to the concise structure of MB-LssDNA-ALP and the simplified detection step without target amplification. These results demonstrated that this platform can be used for high-accuracy analysis.

Finally, the stability of the bioassay components used in this work was verified. The prepared MB-LssDNA-ALP was stored at 4 °C for 16 days. As shown in Figure S8, the signal intensities at different testing times were almost identical, which proved the good storage stability of the biosensing components constructed for CCCB. The robust S/N ratio during the storage period offered a foundation for continuous high-sensitivity analysis with this method. In general, the performance in key areas, including sensitivity, specificity, repeatability, and stability, of the CCCB method for the detection of HPV-16 and PB-19 was verified, which provided a basis for its application in complex biological specimens.

### 3.6. Application of CCCB in Clinical Specimens

**Figure 6:**
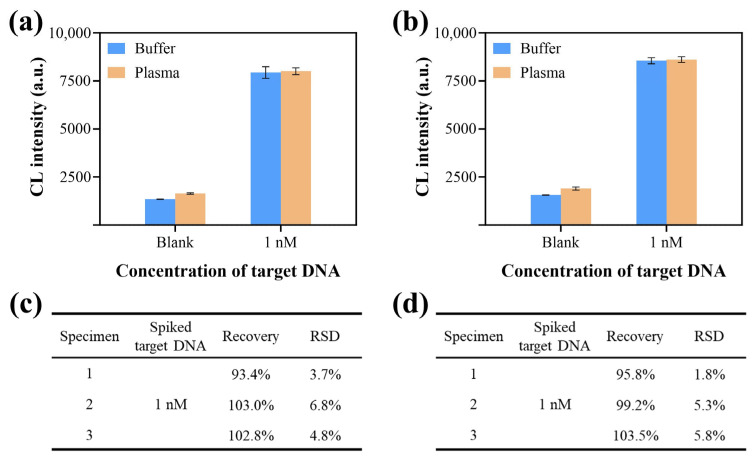
The CCCB strategy for nucleic acid detection in clinical specimens. Signal comparison between buffer and plasma specimens for (**a**) HPV-16 and (**b**) PB-19 testing. Recovery data for plasma specimens spiked with target DNA for (**c**) HPV-16 and (**d**) PB-19 detection.

The practicality of the designed CCCB strategy in complex clinical specimens was investigated by detecting target DNA spiked into 10% human plasma specimens. As shown in Figure 6a,b, the target DNA generated highly consistent CL responses across buffer and plasma samples at matched concentrations, indicating the feasibility of the CCCB platform for detecting real clinical specimens. Furthermore, Figure 6c,d recorded and analyzed the recovery rate data of the bioassay toward target-spiked plasma specimens. In spiked plasma from three individuals, detected target DNA concentrations approximated nominal values with recovery rates of 93.4–103.0% (HPV-16) and 95.8–103.5% (PB-19), implying the practical applicability and reliability of the proposed method for determining HPV-16 and PB-19 in complex clinical biological specimens.

## 4. Conclusions

In summary, an advanced CRISPR/Cas12a-chemiluminescence cascaded bioassay (CCCB) for the amplification-free and sensitive detection of nucleic acids (HPV-16 and PB-19 used in this case) was developed. The enhanced amplification of the nucleic acid signal is achieved through a cascade enzyme reaction, which combines the trans-cleavage activity of Cas12a with the catalytic function of ALP. Through the rational design of the LssDNA sequence, its triple functionality enables the significant structural simplification of the bioassay components. Compared with the traditional single Cas12a-based approach, this cascaded strategy achieves a substantial increase in sensitivity. Meanwhile, this analysis method can identify target sequences with high specificity and exhibit signal responses to the base mutations. This strategy avoids complex instruments and laborious operations with a low risk of contamination. Indeed, compared to well-established PCR technology with target amplification, additional optimization is required to further enhance the sensitivity of the CCCB strategy. Considering the natural double-stranded structure of certain viral nucleic acids, the CCCB method requires further development to overcome its current limitation of solely detecting single-stranded targets. Overall, this CCCB strategy provides a generalizable, cost-effective, and robust nucleic acid analysis tool for the accurate diagnosis of various infectious diseases.

## Data Availability

The data supporting the findings of this study are available within the paper.

## Supplementary Materials

The following supporting information can be downloaded at: https://www.mdpi.com/article/10.3390/bios15080479/s1, Figure S1: Particle size analysis; Figure S2: Standard curve of Cy3-labeled LssDNA; Figure S3: Optimization of analysis conditions for HPV-16 detection; Figure S4: Optimization of analysis conditions for PB-19 detection; Figure S5: The principle of the single Cas12a-based detection system; Figure S6: Standard curve of single Cas12a-based method; Figure S7: Repeatability verification of the CCCB strategy; Figure S8: Stability verification of the bioassay components; Table S1: Oligonucleotides used in this work; Table S2: Comparison among recent amplification-free nucleic acid detection methods. References [13,21,32,34,35,37,38,39,40] are cited in Supplementary Materials.
