# Supplementary material for: CRISPR/Cas12a-Chemiluminescence Cascaded Bioassay for Amplification-Free and Sensitive Detection of Nucleic Acids
Source: Biosensors (Basel). 2025 Jul 24;15(8):479. doi: 10.3390/bios15080479 (PMC12384274; doi:10.3390/bios15080479)
Supplement: Supplementary file 1 [file biosensors-15-00479-s001.zip › biosensors-3719362-supplementary.pdf]

Supplementary Information for

CRISPR/Cas12a-Chemiluminescence Cascaded Bioassay for Amplification-Free and Sensitive Detection of Nucleic Acids

Xiaotian Guan [1,†], Peizheng Wang [1,†], Yi Wang [2] and Shuqing Sun [1,*]

[1] Institute of Biopharmaceutical and Healthcare Engineering, Shenzhen International Graduate School, Tsinghua University, Shenzhen 518055, China; guanxt22@mails.tsinghua.edu.cn (X.G.); wpz21@mails.tsinghua.edu.cn (P.W.)

[2] Experimental Research Center, China Academy of Chinese Medical Sciences, Beijing, 100010, China; wangyi02@tsinghua.org.cn

* Correspondence: sun.shuqing@sz.tsinghua.edu.cn

† These authors contributed equally to this work.

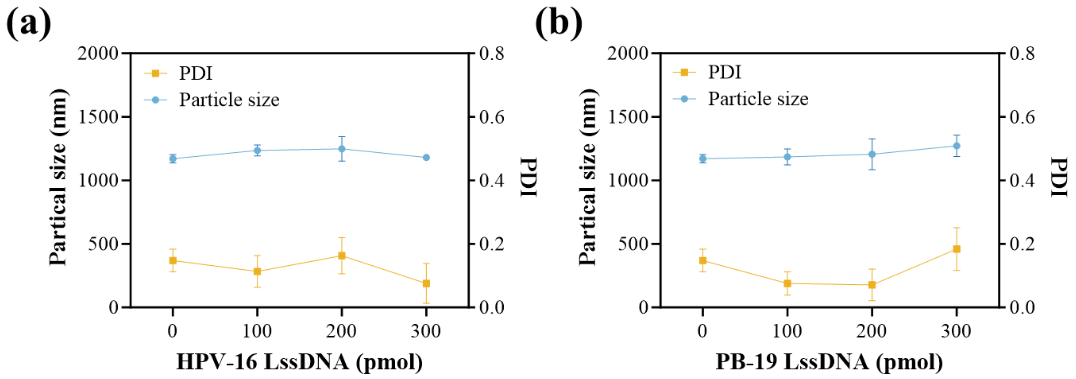

Figure S1. Particle size analysis of (**a**) MB-LssDNA(HPV-16) and (**b**) MB-LssDNA(PB-19) at various synthesis ratios of LssDNA.

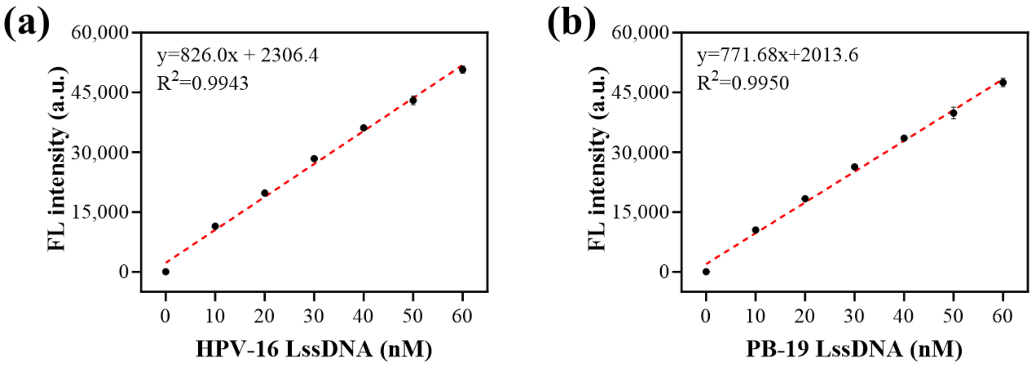

Figure S2. The concentration standard curve of Cy3-labeled (**a**) HPV-16 and (**b**) PB-19 LssDNA ($\lambda_{ex/em}$=535/595 nm).

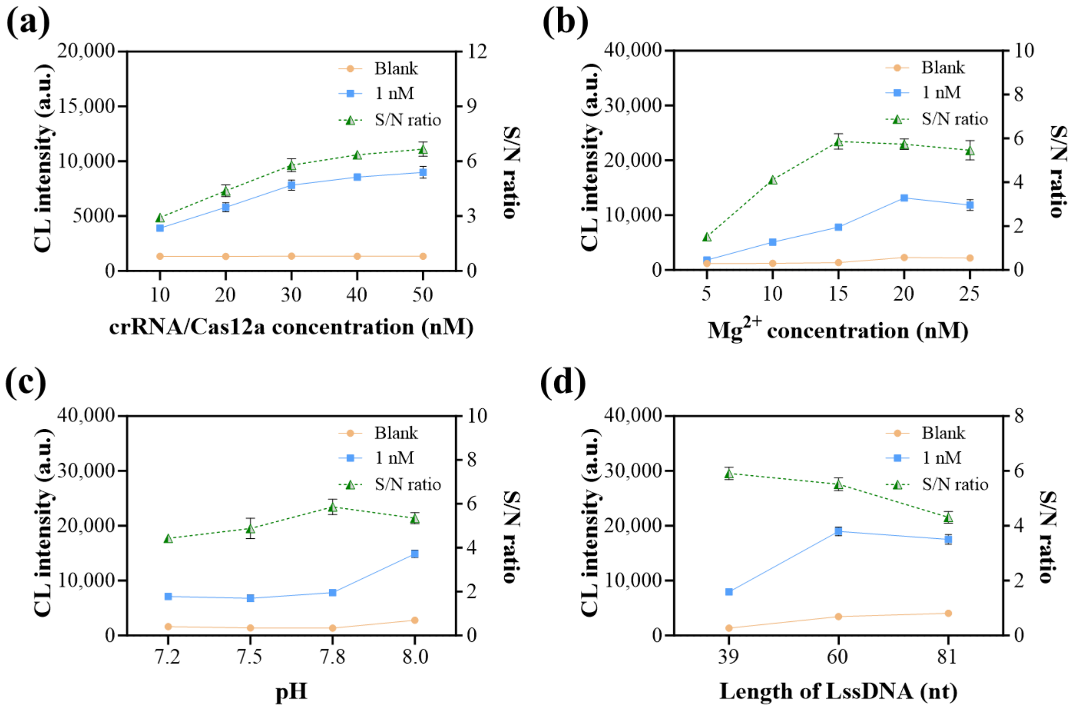

Figure S3. Optimization of analysis conditions for HPV-16 detection. The concentration of (**a**) crRNA/Cas12a and (**b**) Mg^{2+}; (**c**) The pH of Tris-HCl buffer; (**d**) The length of LssDNA sequence.

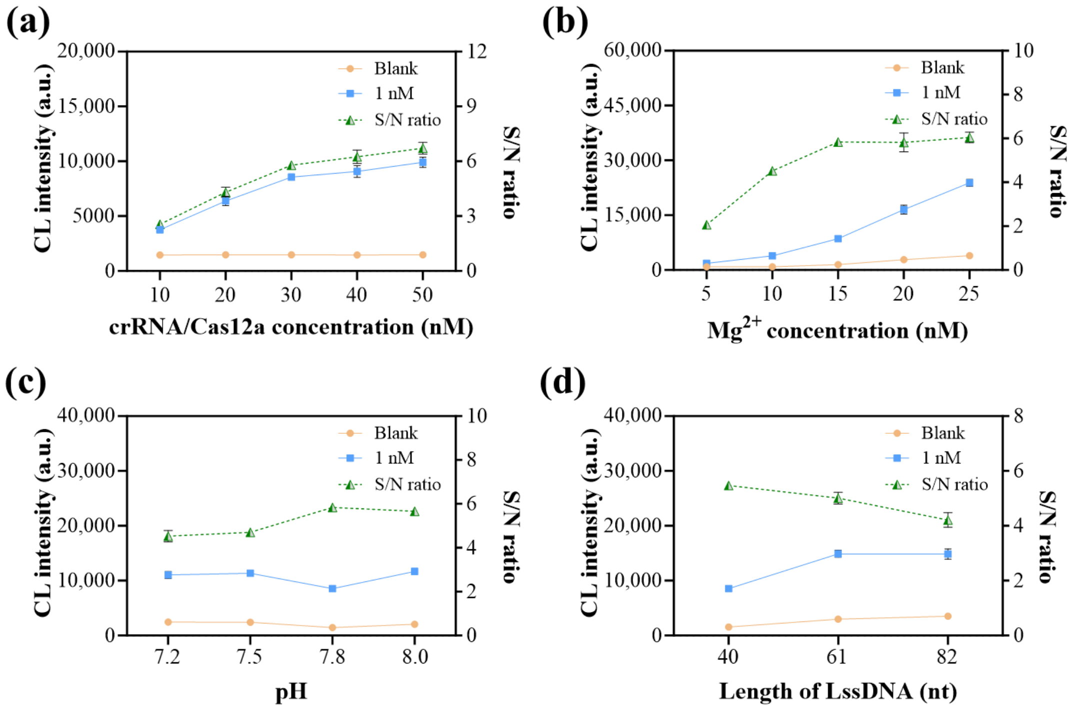

Figure S4. Optimization of analysis conditions for PB-19 detection. The concentration of (**a**) crRNA/Cas12a and (**b**) Mg^{2+}; (**c**) The pH of Tris-HCl buffer; (**d**) The length of LssDNA sequence.

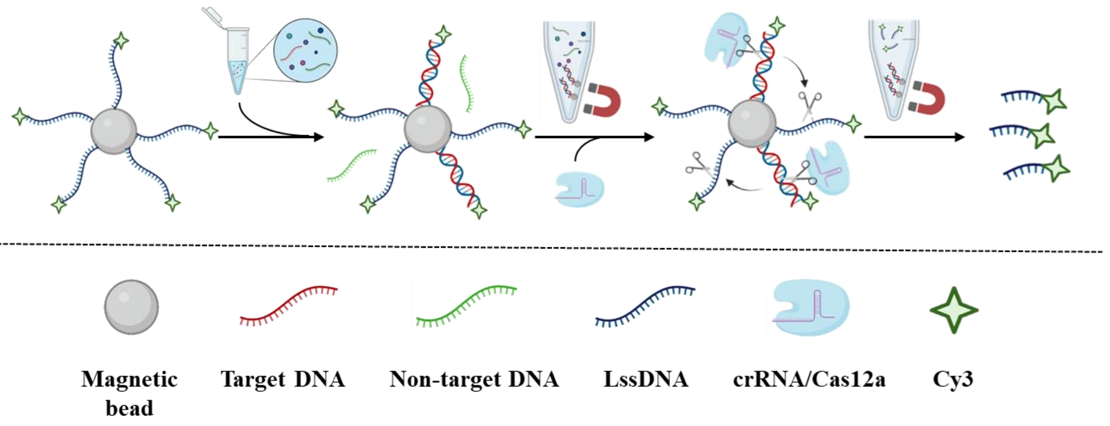

Figure S5. The principle of the single Cas12a-based detection system. The single-stranded target DNA was initially captured and enriched by LssDNA via base hybridization, and the trans-cleavage activity of Cas12a was then activated through sequence recognition between crRNA and target DNA. Following efficient Cas12a-mediated cleavage of LssDNA, Cy3 labeled on LssDNA was released from MB into solution. Finally, the fluorescence signal intensity of Cy3 in the supernatant was measured.

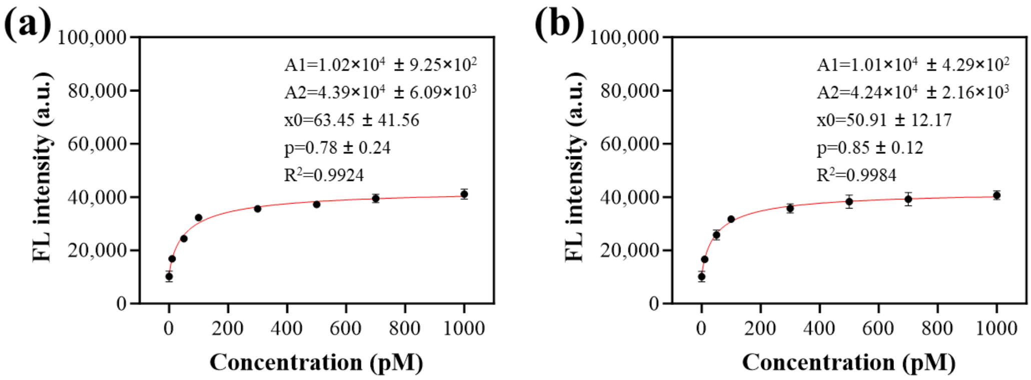

Figure S6. The concentration standard curve of the single Cas12a-based method for (a) HPV-16 and (b) PB-16 detection ($\lambda_{ex/em}$=535/595 nm). The target DNA were 0, 10, 50, 100, 300, 500, 700 and 1000 pM.

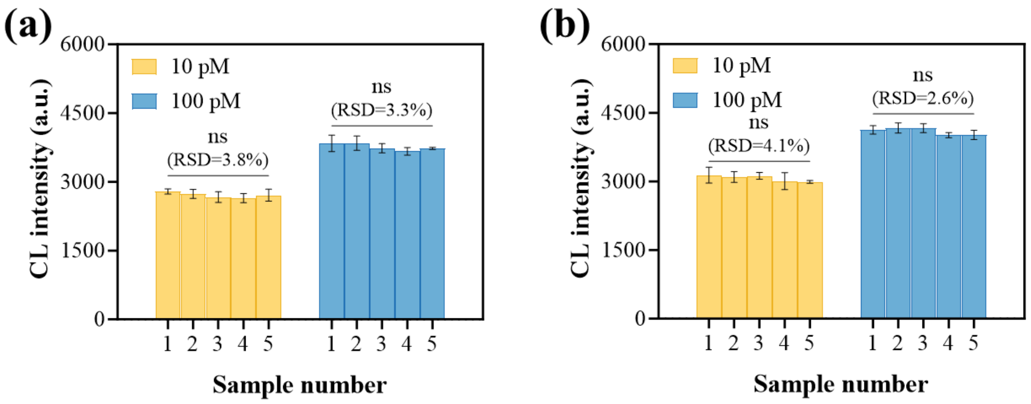

Figure S7. Repeatability verification of the CCCB strategy. Replicate testing was performed five times for identical samples at two concentrations for (**a**) HPV-16 and (**b**) PB-19 detection.

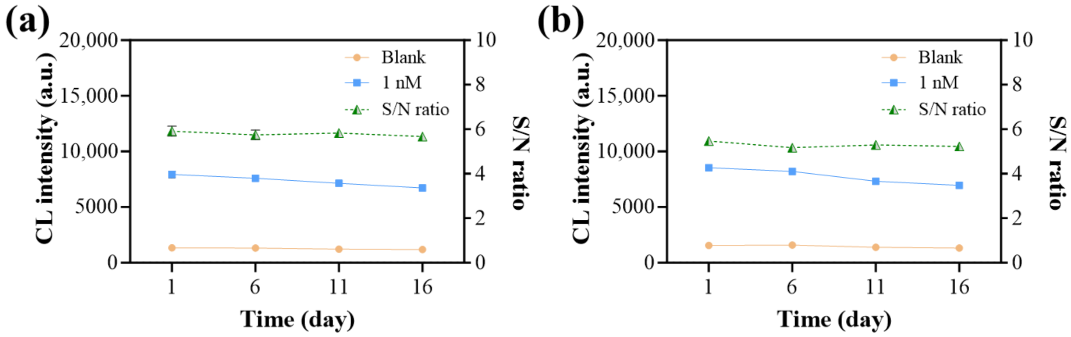

Figure S8. Stability verification of the bioassay components. The (**a**) MB-LssDNA(HPV-16)-ALP and (**b**) MB-LssDNA(PB-19)-ALP were prepared on day 1, and were used for detection on days 1, 6, 11 and 16.

Table S1. Oligonucleotides used in this work.

Name	Sequence (5' - 3')
HPV-16 (target DNA)	AATATGTCATTATGTGCTGCCATATCTACTTCAGAAACT
PB-19 (target DNA)	CATTATTAAGTCCACTATTGTGGAAGCTGCAAAAGCTATT
crRNA(HPV-16)	UAAUUUCUACUAAGUGUAGAUGAAGUAGAUAUGGCAGCAC
crRNA(PB-19)	UAAUUUCUACUAAGUGUAGAUCAGCUUCCACAAUAGUGGAC
Cy3 labled LssDNA(HPV-16)	Cy3-AGTTTCTGAAGTAGATATGGCAGCACATAATGACATATT-NH_2
Cy3 labled LssDNA(PB-19)	Cy3-AATAGCTTTTGCAGCTTCCACAATAGTGGACTTAATAATG-NH_2
LssDNA(HPV-16)-39 nt	Biotin-AGTTTCTGAAGTAGATATGGCAGCACATAATGACATATT-NH_2
LssDNA(HPV-16)-60 nt	Biotin-AGTTTCTGAAGTAGATATGGCAGCACATAATGACATATTCTAGT<br>TACAGCTATCTCATCC-NH_2
LssDNA(HPV-16)-81 nt	Biotin-AGTTTCTGAAGTAGATATGGCAGCACATAATGACATATTCTAGT<br>TACAGCTATCTCATCCCTAGTTACAGCTATCTCATCC-NH_2
LssDNA(PB-19)-40 nt	Biotin-AATAGCTTTTGCAGCTTCCACAATAGTGGACTTAATAATG-NH_2
LssDNA(PB-19)-61 nt	Biotin-AATAGCTTTTGCAGCTTCCACAATAGTGGACTTAATAATGCTAG<br>TTACAGCTATCTCATCC-NH_2
LssDNA(PB-19)-82 nt	Biotin-AATAGCTTTTGCAGCTTCCACAATAGTGGACTTAATAATGCTAG<br>TTACAGCTATCTCATCCCTAGTTACAGCTATCTCATCC-NH_2
HPV-18	AGGTACAGGAGACTGTGTAGAAGCACATATTGTTAAATT
Scrambled	CTAGTTACTGCTATCAGGTCCTCAGTGCCTGCATGTAGA
Mismatch 1 HPV-16	AATATGTCATTATGTGCTGCCATATCTACTTCAGAATCT
Mismatch 2 HPV-16	AATATGTCATTATGTGCTGCCATATCTACTTCTGAAACT
Mismatch 3 HPV-16	AATATGTCATTATGTGCTGCCATATCTTCTTCAGAAACT
Mismatch 4 HPV-16	AATATGTCATTATGTGCTGCCAAATCTACTTCAGAAACT
Mismatch 5 HPV-16	AATATGTCATTATGTGCAGCCATATCTACTTCAGAAACT
Mismatch 1 PB-19	CATTATTAAGTCCACTATTGTGGAAGCTGCAATAGCTATT
Mismatch 2 PB-19	CATTATTAAGTCCACTATTGTGGAAGCTCCAAAAGCTATT
Mismatch 3 PB-19	CATTATTAAGTCCACTATTGTGGTAGCTGCAAAAGCTATT
Mismatch 4 PB-19	CATTATTAAGTCCACTATAGTGGAAGCTGCAAAAGCTATT
Mismatch 5 PB-19	CATTATTAAGTCCTCTATTGTGGAAGCTGCAAAAGCTATT

The PAM sequences were marked with the underlines, and the mismatch positions were highlighted in red.

Table S2. Comparison among recent amplification-free nucleic acid detection methods.

Method	Target	Detection range	LOD	Testing time (min)	Ref.
Cas12a-based electrochemiluminescence biosensor	HPV-16	1 pM-10 nM	0.48 pM	70	[1]
CRISPR/Cas12a-mediated liposome-amplified strategy	HPV-16	5 pM-100 nM	1.6 pM	>55	[2]
CRISPR/Cas12a-based chemiluminescence imaging sensor	HPV-16	10 pM-800 pM	3 pM	250	[3]
CRISPR/Cas12a-based electrochemical biosensor	HPV-16 and PB-19	10 pM-100 nM	50 pM	55	[4]
CRISPR/Cas12a powered DNA framework-supported electrochemical biosensing	HPV-16	100 fM-100 pM	100 fM	195	[5]
Electrochemical biosensor based on Cas12a-mediated interfacial cleaving of hairpin DNA	HPV-16	50 pM-100 nM	30 pM	60	[6]
DNA tetrahedron-based CRISPR bioassay	HPV-16	0.1 pM-50 pM	218 fM	140	[7]
Catalytic hairpin assembly amplification	H1N1	0.5 nM-25 nM	136 pM	20	[8]
Ratiometric fluorometric assay triggered by alkaline phosphatase	H1N1	50 pM-1.5 nM	10 pM	180	[9]
CRISPR/Cas12a-chemiluminescence cascaded bioassay	HPV-16 and PB-19	10 pM-1000 pM	0.14 pM / 0.37 pM	60	This work

References

1. Liu, P.F.; Zhao, K.R.; Liu, Z.J.; Wang, L.; Ye, S.Y.; Liang, G.X. Cas12a-based electrochemiluminescence biosensor for target amplification-free DNA detection. *Biosens. Bioelectron.* **2021**, *176*, 7.
2. Gong, H.X.; Wu, Y.L.; Zeng, R.J.; Zeng, Y.Y.; Liu, X.L.; Tang, D.A.P. CRISPR/Cas12a-mediated liposome-amplified strategy for the photoelectrochemical detection of nucleic acid. *Chem. Commun.* **2021**, *57*, 8977-8980.
3. Ke, X.X.; Ou, Y.J.; Lin, Y.; Hu, T. Enhanced chemiluminescence imaging sensor for ultrasensitive detection of nucleic acids based on HCR-CRISPR/Cas12a. *Biosens. Bioelectron.* **2022**, *212*, 8.
4. Dai, Y.F.; Somoza, R.A.; Wang, L.; Welter, J.F.; Li, Y.; Caplan, A.I.; Liu, C.C. Exploring the Trans-Cleavage Activity of CRISPR-Cas12a (cpf1) for the Development of a Universal Electrochemical Biosensor. *Angew. Chem.-Int. Edit.* **2019**, *58*, 17399-17405.
5. Su, J.; Ke, Y.Q.; Maboyi, N.; Zhi, X.; Yan, S.J.; Li, F.W.; Zhao, B.; Jia, X.L.; Song, S.P.; Ding, X.T. CRISPR/Cas12a Powered DNA Framework-Supported Electrochemical Biosensing Platform for Ultrasensitive Nucleic Acid Analysis. *Small Methods* **2021**, *5*, 11.
6. Zhang, D.C.; Yan, Y.R.; Que, H.Y.; Yang, T.T.; Cheng, X.X.; Ding, S.J.; Zhang, X.M.; Cheng, W. CRISPR/Cas12a-Mediated Interfacial Cleaving of Hairpin DNA Reporter for Electrochemical Nucleic Acid Sensing. *ACS Sens.* **2020**, *5*, 557-562.
7. Zhan, X.H.; Zhou, J.; Jiang, Y.J.; An, P.; Luo, B.; Lan, F.; Ying, B.W.; Wu, Y. DNA tetrahedron-based CRISPR bioassay for treble-self-amplified and multiplex HPV-DNA detection with elemental tagging. *Biosens. Bioelectron.* **2023**, *229*, 9.
8. Jiang, D.G.; Tian, Y.F.; Zhang, Y.J.; Lu, X.Y.; Xiao, D.; Zhou, C.S. One-step fast and label-free imaging array for multiplexed detection of trace avian influenza viruses. *Anal. Chim. Acta* **2021**, *1171*, 7.
9. Chen, L.G.; Li, J.J.; Sun, L.; Wang, H.B. Ratiometric fluorometric assay triggered by alkaline phosphatase: Proof-of-concept toward a split-type biosensing strategy for DNA detection. *Talanta* **2024**, *271*, 8.
